# Crystal structure of dipeptidyl peptidase III from the human gut symbiont *Bacteroides thetaiotaomicron*

**DOI:** 10.1371/journal.pone.0187295

**Published:** 2017-11-02

**Authors:** Igor Sabljić, Nevenka Meštrović, Bojana Vukelić, Peter Macheroux, Karl Gruber, Marija Luić, Marija Abramić

**Affiliations:** 1 Division of Physical Chemistry, Ruđer Bošković Institute, Zagreb, Croatia; 2 Division of Molecular Biology, Ruđer Bošković Institute, Zagreb, Croatia; 3 Division of Organic Chemistry and Biochemistry, Ruđer Bošković Institute, Zagreb, Croatia; 4 Institute of Biochemistry, Graz University of Technology, Graz, Austria; 5 Institute of Molecular Biosciences, University of Graz, Graz, Austria; University of Queensland, AUSTRALIA

## Abstract

*Bacteroides thetaiotaomicron* is a dominant member of the human intestinal microbiome. The genome of this anaerobe encodes more than 100 proteolytic enzymes, the majority of which have not been characterized. In the present study, we have produced and purified recombinant dipeptidyl peptidase III (DPP III) from *B*. *thetaiotaomicron* for the purposes of biochemical and structural investigations. DPP III is a cytosolic zinc-metallopeptidase of the M49 family, involved in protein metabolism. The biochemical results for *B*. *thetaiotaomicron* DPP III from our research showed both some similarities to, as well as certain differences from, previously characterised yeast and human DPP III. The 3D-structure of *B*. *thetaiotaomicron* DPP III was determined by X-ray crystallography and revealed a two-domain protein. The ligand-free structure (refined to 2.4 Å) was in the open conformation, while in the presence of the hydroxamate inhibitor Tyr-Phe-NHOH, the closed form (refined to 3.3 Å) was observed. Compared to the closed form, the two domains of the open form are rotated away from each other by about 28 degrees. A comparison of the crystal structure of *B*. *thetaiotaomicron* DPP III with that of the human and yeast enzymes revealed a similar overall fold. However, a significant difference with functional implications was discovered in the upper domain, farther away from the catalytic centre. In addition, our data indicate that large protein flexibility might be conserved in the M49 family.

## Introduction

*Bacteroides thetaiotaomicron* is one of the best studied representatives of the *Bacteroides* spp, a group of anaerobic, Gram-negative microbes, which are part of the human intestinal microbiome and play a central role in symbiotic host-bacterial relationships in the human gut [1]. These bacteria are known for their ability to degrade a wide variety of glycans that are not substrates for human glycosidases. The completed genome sequence of *B*. *thetaiotaomicron* revealed that a large fraction of the 4779 encoded proteins participate in harvesting otherwise indigestible dietary polysaccharides and in metabolizing their liberated sugars (*e*.*g*. 172 glycosyl hydrolases, 163 homologues of SusC and SusD outer membrane polysaccharide-binding proteins; 20 sugar-specific transporters) [2]. The genome sequence also provided insight into the proteolytic capacity of *B*. *thetaiotaomicron*: according to the *MEROPS* database (http://merops.sanger.ac.uk/; release 11.0) [3], this bacterium possesses 129 peptidases. The majority of these peptidases have not been functionally or biochemically characterized.

Recently, we have cloned and biochemically characterized dipeptidyl peptidase III (DPP III) from *B*. *thetaiotaomicron* [4]. DPP III (EC 3.4.14.4; earlier names: dipeptidyl arylamidase III, dipeptidyl aminopeptidase III) is a cytosolic zinc-metallopeptidase of the M49 family [3]. This exopeptidase catalyses the hydrolysis of the penultimate peptide bond of its oligopeptide substrates with unsubstituted N-termini, thereby sequentially removing N-terminal dipeptides [5, 6]. It is broadly distributed in eukaryotic cells and considered to participate in normal intracellular protein catabolism. In addition to its proteolytic activity, the mammalian enzyme appears to be involved in cellular defence against oxidative stress as an activator in the Keap1-Nrf2 signalling pathway [7, 8].

The M49 family of metallopeptidases (DPP III family) is defined by five conserved amino acid sequence regions, including the unique hexapeptide zinc-binding motif, HEXXGH [9, 10]. A second conserved motif involved in the coordination of the active-site zinc, EEXR(K)AE(D), is situated 22–55 amino acids toward the C-terminus from the first one [10]. Until now, three-dimensional structures of two eukaryotic DPP III enzymes have been solved: yeast and human DPP III, respectively [11, 12]. The crystal structures of ligand-free human and yeast enzymes revealed an elongated protein molecule with two domains separated by a wide cleft, as well as a very similar overall fold. The X-ray structure of human DPP III in complex with the pentapeptide tynorphin showed that ligand binding was accompanied by a large domain motion and a closure of the inter-domain cleft [12].

*In silico* analyses of the amino acid sequences of the M49 family peptidases revealed low similarity of the bacterial sequences with the eukaryotic ones. In all eukaryotic DPPs III, the second zinc-binding motif contained a cysteine residue (EECRAE), while the majority of the bacterial sequences contained a cysteine in the first motif (HECLGH) [4]. In order to investigate the properties of the bacterial orthologues of the M49 family, we cloned and heterologously expressed the cDNA encoding full-length DPP III from *B*. *thetaiotaomicron* (*Bt*DPP III, 675 amino acids), biochemically characterized the purified recombinant protein and compared it to the human enzyme [4]. *Bt*DPP III is a monomeric acidic protein (Mr ~ 76000, pI ~5.1) which hydrolyses the preferred synthetic substrate of mammalian DPPs III, Arg-Arg-2-naphthylamide (Arg_2_-2NA), with a pH optimum of 8.0. The hydrolytic activity of purified *Bt*DPP III is, similarly to that of the eukaryotic enzymes, enhanced when Co^2+^ ions are added to the reaction mixture, and abolished in the presence of chelating agents and sulfhydryl reagents [4]. Compared to its human counterpart, the bacterial enzyme differed in its pHhH optimum and kinetic parameters for Arg_2_-2NA hydrolysis (3-fold lower *K*_m_ value and 6-fold lower *k*_cat_ value determined with *Bt*DPP III). Furthermore, we observed some differences in the inhibitory potency of novel dipeptidyl hydroxamic acids [13].

We performed this study to gain insight into the three-dimensional structure of *Bt*DPP III, to be able to compare it with its human counterpart and to establish the structural determinants of the similarities/differences between them. Aside from its symbiotic role, *B*. *thetaiotaomicron* is also known as an opportunistic pathogen, which is of clinical interest [4]. In general, *Bacteroides* species are significant clinical pathogens when they escape the gut environment. We propose that DPP III is involved in protein metabolism in *B*. *thetaiotaomicron* and in many other *Bacteroides* species and that it contributes to the growth of these bacteria. In addition, the aim of this study was to find out whether large protein flexibility is conserved in the DPP III family. Here, we describe for the first time the three-dimensional structure of the bacterial orthologue of the M49 family in both open and closed conformations. Our results revealed that the overall protein fold is very similar to that of the human and yeast orthologue, with two domains separated by a wide cleft containing a catalytic zinc ion. However, significant structural differences in the bacterial protein were observed in both the upper and the lower domain.

## Materials and methods

### Cloning and site-directed mutagenesis

The *BT_1846* gene encoding DPP III enzyme was amplified from the genomic DNA of *B*. *thetaiotaomicron* using PCR with the primers listed in S1 Table. The PCR product was cloned into *Nhe*I and *Xho*I sites of the pET-21b(+) vector, resulting in a construct containing a hexa-histidine tag (-LEHHHHHH) at the C-terminal end of the enzyme. Point mutations of the enzyme were carried out with the QuikChange II XL Site-Directed Mutagenesis kit (Agilent Technologies) according to the manufacturer’s instructions. The primers designed to introduce C11S, C158S, C189S, C425S, and C450S are listed in S1 Table. Cys-null was prepared by introducing the point mutations stepwise, starting with C11S.

### Enzyme expression

For heterologous expression of wild-type and Cys-null DPP III, *Escherichia coli* BL21-CodonPlus (DE3)-RIL cells were transformed with the appropriate expression vector (encoding the wild-type or the Cys-null mutant). Further procedure was performed as described for the human DPP III [14], with the exception that, after inducing expression, the culture was grown at 37°C for 4 h. Bacterial cells were harvested by centrifugation at 5000 g at 4°C for 20 minutes and stored at -20°C until purification.

DPP III labelled with selenomethionine (Se-Met) was produced by transforming *Escherichia coli* B834(DE3) cells with the Cys-null construct. One of the transformed colonies was inoculated into 10 ml of Luria broth medium supplemented with 100 μg·mL^-1^ ampicillin and was then grown overnight. The following day, 10 mL of the overnight cell culture was added into 0.5 L of minimal medium containing 7.5 mM (NH_4_)_2_SO_4_, 8.5 mM NaCl, 22 mM KH_2_PO_4_, 50 mM K_2_HPO_4_, 1 mM MgSO_4_, 20 mM D-glucose monohydrate, 1 μg·mL^-1^ CaCl_2_, 1 μg·mL^-1^ FeCl_2_, 0.01 μg·mL^-1^ of trace elements (CuSO_4_, ZnCl_2_, MnCl_2_ and (NH_4_)_2_MoO_4_), 10 μg·mL^-1^ thiamine, 10 μg·mL^-1^ biotin, 100 μg·mL^-1^ ampicillin, 50 mg·L^-1^ of all amino acids except methionine, and 40 μM methionine. The cells were grown for 8–10 h at 37°C and 150 rpm until *D*_600nm_ reached a constant value. At that point, all methionine was depleted, and incubation was continued without methionine under the same conditions for two more hours. Protein expression was induced with 0.5 mM isopropyl thio-β-D-galactoside, and 0.125 mM selenomethionine was added to the cell culture. The growth was continued for 3–4 h, before harvesting the cells by centrifugation at 5000 g for 20 minutes [15].

### Enzyme purification

All of the following procedures were performed at 4°C. The cells from 2–4 L of culture were resuspended in up to 50 mL of lysis buffer (5 mL solution per 1 g of pellet), containing 50 mM Tris-HCl (pH 8.0), 300 mM NaCl, and 10 mM imidazole. The cell suspension was lysed by sonication and then centrifuged for 45 minutes at 14500 g. The supernatant was filtrated using Rotilabo-syringe filters with 0.45 μm cut-off (ROTH, Karlsruhe, Germany) to remove all remaining cell debris and was then applied for affinity chromatography on Ni-NTA resin (5 mL pre-packed His-trap FF, GE Healthcare) fitted in an ÄKTA FPLC (GE Healthcare) that had been equilibrated with the lysis buffer. The affinity column was washed with 25 mL of the lysis buffer. Protein elution was performed using a 50-mL linear gradient of 10–500 mM imidazole in the same buffer. The obtained enzyme sample was then applied to Superdex 200 (26/60 or 16/60, depending on the sample volume) gel filtration column (GE Healthcare) previously equilibrated with 50 mM Tris-HCl (pH 7.4) containing 100 mM NaCl. Fractions with purified protein corresponding to a molecular mass of ~77 kDa were collected and concentrated using centrifugal filtration (Amicon 10K; Millipore, Bedford, MA, USA). The purity of the fractions was analysed by SDS-PAGE (12% gel), and the protein concentration was determined by measurement at *A*_280_ nm, using the theoretical molar extinction coefficient, 99130 M^-1^·cm^-1^, determined by ProtParam tool on ExPASy SIB Bioinformatics Resource Portal [16]. The homogeneity of the fractions was analysed by isoelectric focusing (IEF) on PhastGel IEF plates with pH gradient 4–6.5 (GE Healthcare). For long-term storage, the purified enzyme was kept at –80°C.

### Determination of kinetic parameters

The release of the fluorescent product (2-naphthylamine, 2NA) of enzymatic hydrolysis of dipeptidyl-2-naphthylamides was used for the initial rate measurements as described by Abramić et al. [5], and kinetic parameters (*K*_m_ and *k*_cat_) were determined by non-linear regression, using GraphPad Prism 7.03. Enzymatic reactions were performed at 25°C and at pH 8.0 (20 mM Tris-HCl), with the dipeptidyl-2-naphthylamide substrate (0.05 μM to 5 μM Arg_2_-2NA, 0.6 μM to 40 μM Ala–Arg-2NA, or 0.6 μM to 80 μM Phe-Arg-2NA), and 0.4 nM (with Arg_2_-2NA and Ala-Arg-2NA) or 1.3 nM (with Phe-Arg-2NA) wild-type *Bt*DPP III. The enzyme was preincubated for 2 minutes in 3-mL reaction mixture, and then the reaction started with the addition of a few microliters of substrate stock solution. Continuous measurement of the fluorescence of the free 2NA was performed for 1 minute by the Agilent Cary Eclipse fluorescence spectrophotometer (emission wavelength 420 nm, slit width 5 nm; excitation wavelength 332 nm, slit width 10 nm). Enzymatic reactions obeyed the Michaelis-Menten kinetics (S2 Fig).

### Phylogenetic analysis

Databases of bacteria, fungi, nematodes, arthropods, and vertebrates from the UniProt repository were analysed for DPP III homologues using the BLAST search tool with *B*. *thetaiotaomicron*, yeast, and human counterparts (UniProt KB entries: Q8A6N1, Q08225, and Q9NY33). Since the databases contained large numbers of homologues, 87 members of the M49 family were selected for the construction of the phylogenetic tree. The selected sequences comprise commonly used model organisms together with the representative sequence from each phylum; 19 bacteria, 10 fungi, 11 nematodes, 10 arthropods and 37 vertebrate homologues. The full names of all species with protein accession numbers are given in S2 Table. Multiple alignment of 87 DPP III protein sequences was performed using ClustalW [17]. The maximum likelihood (ML) tree based on the ClustalW alignment was obtained with the PhyML 3.0 using JTT amino acid substitution model [18]. The phylogenetic tree was displayed with FigTree v1.40 [19] and adjusted in CorelDRAW 12 software [20].

### Crystallization and data collection

Crystallization was done using an Oryx8 robot (Douglas Instruments, UK) by vapour diffusion in sitting drops by mixing 0.5 μL of protein solution and 0.5 μL of crystallization solution at 20°C. Six different commercial screens were used: Midas, JCSG+, PGA, and Morpheus from Molecular Dimensions (Newmarket, UK), the PACT Suite from Qiagen (Hilden, Germany), and Index from Hampton Research (California, USA). The first crystals were obtained in 0.2 M ammonium acetate, 0.1 M MES pH 6.5, 30% v/v glycerol ethoxylate (Midas G7) using Cys-null protein concentrated to 16.6 mg·mL^-1^. Se-Met labelled Cys-null protein concentrated to 28 mg·mL^-1^ produced microcrystals in 0.2 M ammonium chloride, 0.1 M HEPES pH 7.5 and 25% v/v glycerol ethoxylate (Midas H4). These microcrystals were used to prepare a seed stock solution. The seed stock and the working solution were prepared with Seed Bead (Hampton Research) as per the manufacturer’s protocol. Further screening was done by mixing 0.5 μL of protein solution and 0.5 μL of crystallization solution and 0.25 μL of seeding working solution (20x diluted seed stock solution).

Crystals of Se-Met labelled Cys-null protein were obtained in several new conditions: 0.2 M ammonium acetate, 0.1 M MES pH 6.5, 30% glycerol ethoxylate (Midas G7), 0.2 M NaCl, 0.1 M sodium cacodylate pH 6.5, 2.0 M (NH_4_)_2_SO_4_ (JCSG+ E2), 0.1 M bicine, 10% w/v PEG 20000, 2% v/v dioxane pH 9.0 (JCSG+ C10), and 0.1 M Bis-Tris pH 5.5, 2.0 M (NH_4_)_2_SO_4_ (JCSG+ G11). The best diffracting crystals were grown in Midas G7 condition. For the crystallization of the wild-type, the protein sample was concentrated to 18.5 mg·mL^-1^ and a few crystals were obtained in the JCSG+ E2 condition.

We tried to crystallize the complexes of all prepared protein constructs with Tyr-Phe-NHOH. This compound was chosen because it is a substrate analogue of DPP III, previously shown to be a potent competitive inhibitor of *Bt*DPP III [13]. The inhibitor was dissolved in the same buffer as the protein and mixed in an approximately 1:30 protein:inhibitor molar ratio, after which it was incubated for 20 minutes at 4°C. This mixture was used in crystallization trials with four known crystallization conditions, as described for Se-Met labelled Cys-null protein. One crystal was obtained with Se-Met labelled Cys-null DPP III in the JCSG+ E2 condition. Despite our extensive efforts, we were not able to reproduce the crystallization of the protein in either the open or the closed conformation.

The crystals were flash-cooled with liquid N_2_, and all of the diffraction experiments were carried out at 100 K at Elettra Sincrotrone Trieste (Trieste, Italy) with a PILATUS 2M detector. The single-wavelength anomalous diffraction (SAD) experiment with Se-Met labelled Cys-null crystal was performed at 0.9718 Å wavelength. We collected 720 images, covering 360° at a resolution of 1.90 Å. Datasets for the wild-type and the structure in the closed form were collected at 0.976 Å wavelength, at resolutions of 2.40 and 3.29 Å, respectively. Data collection and refinement statistics are summarized in Table 1.

**Table 1:** Data collection, structure determination, and refinement statistics^a^.

	Wild-type	Cys-null	Structure in the closed form
Crystal parameters			
Resolution (Å)	2.4	1.9	3.29
Space group	*P*3_1_21	*P*3_1_21	*P*2_1_2_1_2
Unit cell parameters			
*a*, *b*, *c* (Å)	103.5, 103.5, 141.0	103.4, 103.4, 141.2	123.6, 176.3, 74.1
*α*, *β*, *γ* (°)	90.0, 90.0, 120	90.0, 90.0, 120	90, 90, 90
Matthews coefficient (Å^3^/Da)	2.81	2.83	2.62
Solvent content (%)	56.4	56.5	53.0
Data collection			
Completeness (%)	99.9 (99.5)	99.7 (96.6)	99.0 (87.1)
No. of unique reflections	34694	69127	25217
*I*/σ(*I*)	8.4 (2.8)	15.7 (2.5)	7.8 (3.0)
*R*_merge_	0.161 (0.594)	0.087 (0.724)	0.229 (0.592)
CC_1/2_	0.99 (0.822)	0.998 (0.846)	0.974 (0.871)
Redundancy	5.30 (5.50)	6.90 (6.70)	5.30 (4.50)
Wilson B-factor (Å^2^)	25.3	25.8	47.3
Refinement			
*R* / *R*_free_	0.202/0.244	0.173/0.200	0.207/0.251
RMSD bonds length (Å)	0.005	0.008	0.002
Average *B* factor	29.3	31.3	41.0
Number of molecules in AU	1	1	2
No. of atoms			
Protein	5173	5154	5100/5098
Zinc ion	1	1	2
Water molecules	382	575	0
Ramachandran analysis^b^			
Favoured (%)/*n*	98	98	98
Allowed (%)/*n*	2	2	2
Outlier (%)/*n*	0	0	0

The abbreviations RMSD and AU stand for root-mean-square deviation and asymmetric unit, respectively.

^a^ Data for the highest resolution shell are given in parentheses.

^b^ Defined by validation program MOLPROBITY [21].

### Phasing, model building, and refinement

Data processing was performed with XDS [22], and data scaling with Aimless [23] within the CCP4 software suite [24]. Randomly selected 5% of reflections were excluded from all refinements and used to calculate *R*_free_.

Initial single-wavelength (0.9718 Å) anomalous diffraction phases for the Se-Met labelled Cys-null DPP III were obtained with SHELX [25]. SHELXD found nine selenium atoms in the asymmetric unit. These atoms were then used to determine the initial phases, and a poly-Ala model was built using the SHELXE program. The electron density map was further improved using the *Parrot* program [26] within the CCP4 software suite [24]. BUCCANEER software [27] was used to build an initial Cys-null DPP III model. This model was further refined using the programs REFMAC [28, 29] and PHENIX [30]. The COOT program [31] was used for model fitting and real space refinement using *σ*_A_-weighted 2*F*_o_-*F*_c_ and *F*_o_-*F*_c_ electron density maps. Translation, rotation, and screw-rotation (TLS) parameterization of anisotropic displacement was used in the last refinement step [32]. Four TLS groups were defined: 24–328, 329–414, 415–623, and 624–675.

The structures of the wild-type protein and the protein in the closed form were determined by molecular replacement using the MOLREP program [33] within the CCP4 software suite [24], employing the structure of Cys-null DPP III as the model structure. The refinement procedure was the same as for the Cys-null structure, except that no TLS refinement was used for the lower resolution structure that was in the closed form. Structure determination and refinement statistics are given in Table 1. The final coordinates and structure factors have been deposited in the Protein Data Bank (accession number for the wild-type, structure in the closed form and the Cys-null are 5NA7, 5NA8 and 5NA6, respectively).

## Results and discussion

### Protein sample preparation and crystallization

**Fig 1:**
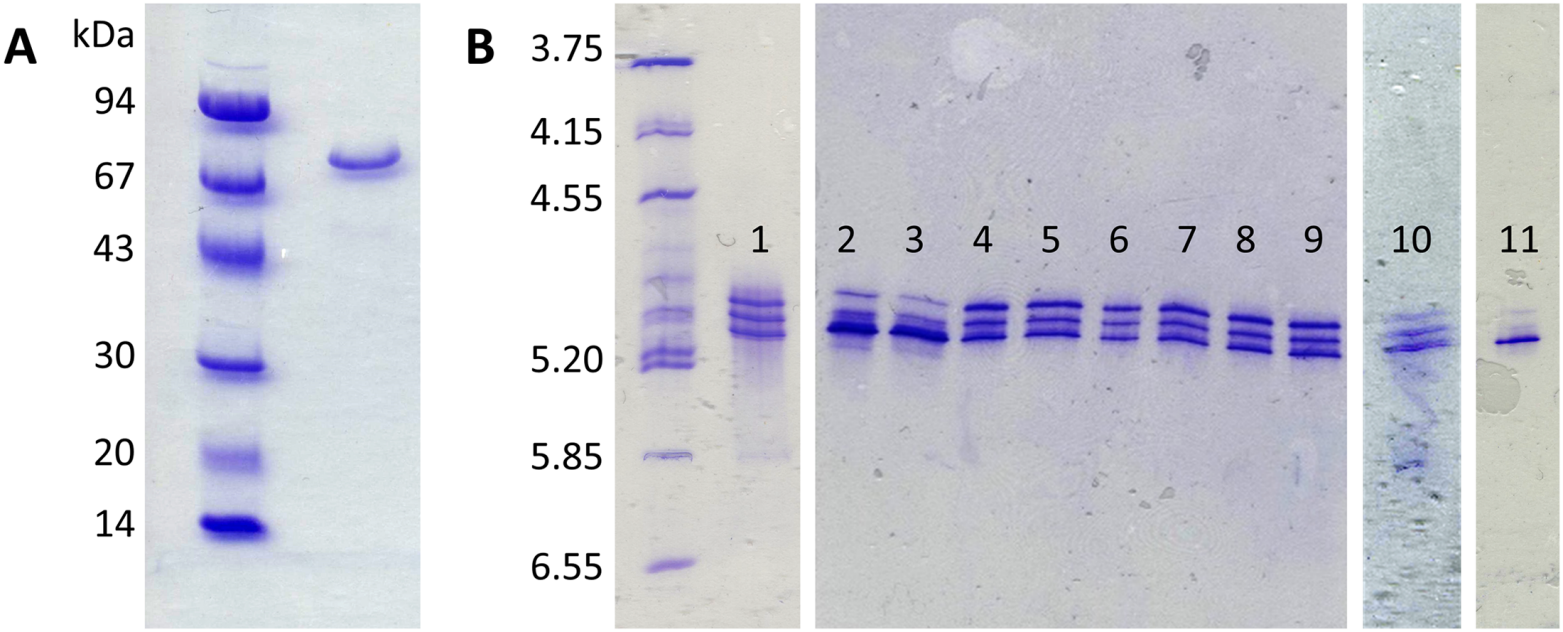
SDS-PAGE and isoelectric focusing analysis of the purified *Bt*DPP III protein variants. (A) SDS-PAGE of purified recombinant wild-type DPP III; (B) Isoelectric focusing analysis of purified wild-type (lane 1) and cysteine variants: C11S (lanes 2–3), C158S (lanes 4–5), C189S (lanes 6–7), C425S (lanes 8–9), C450S (lane 10) and Cys-null (lane 11). Proteins were visualized by Coomassie Blue staining.

Wild-type *Bt*DPP III was produced in *Escherichia coli* and purified employing Ni-NTA affinity chromatography, as described under “Materials and methods”. The purified wild-type protein was analysed using SDS-PAGE and IEF. While SDS-PAGE exhibited a single protein band (Fig 1A), IEF showed three bands (Fig 1B, position 1). Because the protein contains five cysteine residues, we suspected that the oxidation of the side chain thiol group could be the reason behind the observed heterogeneity. The oxidation of a cysteine thiol group results in the formation of a sulfenic, sulfinic or sulfonic acid derivative, leading to additional negative charges. Since we failed to obtain crystals from the purified wild-type protein, we assumed that the heterogeneity introduced by the oxidation of thiol groups had impeded crystallization. In order to identify the cysteine(s) responsible for the charge heterogeneity, we substituted the cysteine residues with serine by site-directed mutagenesis and subsequently purified the recombinant proteins. The five variants, *i*.*e*. C11S, C158S, C189S, C425S, and C450S were subjected to IEF (Fig 1B, lanes 2–10). It was observed that sample C11S is less heterogenic than the wild-type, showing one form in higher concentration, but all three forms were still present (Fig 1B, lanes 2 and 3). Also, a slight difference was observed in the case of C450S, where two forms were present in higher concentrations (Fig 1B, lane 10). The other variants were comparable to the wild-type protein. Thus, our results suggested that the observed charge heterogeneity in the purified *Bt*DPP III was not caused by the oxidation of a single thiol group, but rather the outcome of oxidative modification of several thiol groups. Therefore, we prepared an additional variant with all five cysteine residues replaced by serine (Cys-null variant). As shown in Fig 1B (lane 11) this variant showed significantly improved homogeneity with one dominant protein form (Fig 1B, position 11).

Initial crystallization screening was done using the wild-type protein. As no crystals were obtained, the same crystallization screening was done with a more homogeneous Cys-null protein sample. The first crystals of the Cys-null protein were obtained in the G7 condition of the Midas screen. Microcrystals of the Se-Met labelled Cys-null protein were obtained in the H4 condition of the Midas screen (Molecular Dimensions, Newmarket, UK) and were then used to prepare the seed solution. Further crystallization screens were prepared using microseeding, and crystals of the Se-Met-labelled Cys-null protein were obtained in four new conditions: JCSG+ C10, E2, G11, and Midas G7. The best diffracting crystal was used for the complete data collection, and the crystal structure was solved using SAD at 1.9 Å resolution. The wild-type DPP III crystals were obtained only after the initial crystallization conditions with Se-Met labelled Cys-null protein were determined. Out of more than 100 drops prepared using these four conditions, just a few crystals of the wild-type protein grew in the E2 condition of the JCSG+ screen (Molecular Dimensions, Newmarket, UK), while other drops did not yield any crystals. The structure was solved at 2.4 Å resolution using molecular replacement, with the structure of the Cys-null variant as a search model. Both proteins crystallized in space group *P*3_1_21, with one molecule in the asymmetric unit. By superimposing the wild-type on the Cys-null structures, we confirmed that the replacement of cysteine residues or the incorporation of Se-Met did not change the protein structure (backbone RMSD 0.15 Å). Therefore, only the wild-type structure was considered in further discussion. To obtain a complexed structure, we used the potent competitive inhibitor Tyr-Phe-NHOH. We succeeded in growing only one crystal in the presence of Tyr-Phe-NHOH. This crystal belonged to space group *P*2_1_2_1_2, with two protein molecules in the asymmetric unit. The structure was solved at 3.3 Å resolution using molecular replacement, with the separated structural domains of the Cys-null protein as a model. It was not possible to locate the inhibitor molecule in the electron density maps, but since the conformation changed from open (wild-type) to closed, this structure was included in further consideration.

### Crystal structure of *Bt*DPP III and its comparison with its eukaryotic counterparts

**Fig 2:**
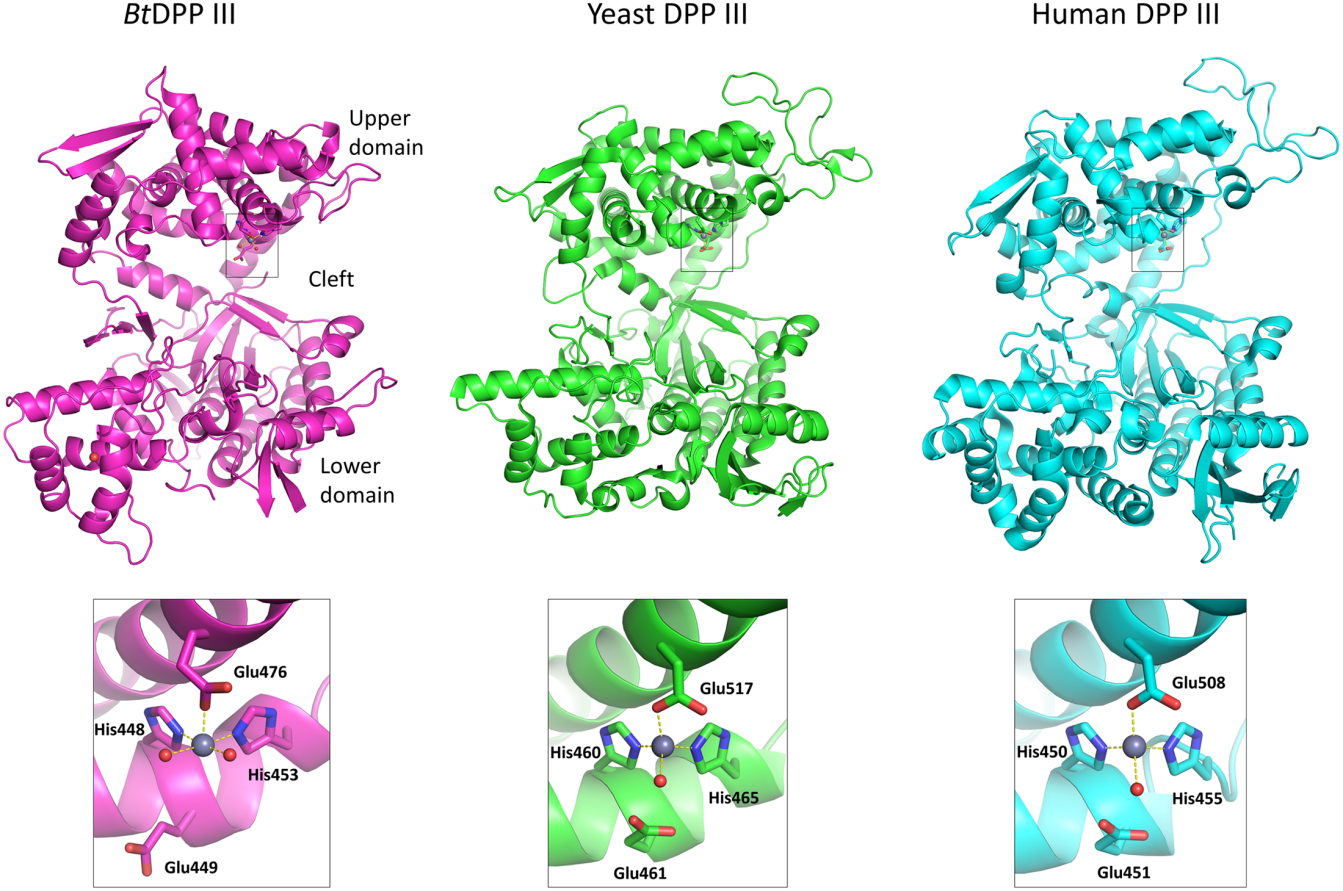
Structures of ligand-free *Bt*, yeast and human DPP III with their respective zinc binding sites. Zinc binding sites are shown in grey squares. Amino acids coordinating the zinc ions (shown as grey spheres) and the glutamic acid residues essential for enzyme activity are shown in stick representations. The figure was prepared using the PyMol program (http://www.pymol.org/), and the PDB-deposited crystal structures of the yeast (PDB ID 3csk) and human DPP III (PDB ID 3fvy).

Despite a low sequence identity (17–21%), the overall structure of *Bt*DPP III is very similar to the previously reported crystal structures of human and yeast DPP III [11, 12] and consists of two domains separated by a wide cleft (Fig 2). The upper structural domain (C-terminal) is mostly helical, while the lower one contains mixed secondary structural elements with a five-stranded β-barrel core (Fig 2). A search for similar folds, performed by structure alignment using PDBeFold [34], did not yield any other matches except for the already known structures of yeast and human DPP III. The catalytic zinc ion is positioned in the lower part of the upper structural domain, coordinated by His448, His453, and Glu476. The two histidine residues are part of the conserved motif HEXXGH of the M49 family, and Glu476 is part of the second active site motif EEXR(K)AE(D). Although the zinc ion identity was not confirmed experimentally in *Bt*DPP III structures, zinc was the most likely candidate as a catalytic ion for several reasons. Firstly, zinc content was previously determined in DPP III purified from human placenta and in the recombinant rat enzyme (expressed in *E*. *coli*) by atomic absorption spectrometry, revealing that DPP III contains 1 mole of zinc per mole of protein [9]. Furthermore, with site-directed mutagenesis of rat DPP III and zinc content determination in the produced DPP III protein variants, three amino acids that coordinate the active-site zinc ion were determined [35]. Those were His450 and His455 from the HELLGH motif, and Glu508 from the EECRAE motif. Additionally, the zinc binding site in DPP III crystal structures resembles the zinc binding site of many other zinc-peptidases, including thermolysin and neprilysin [11]. The coordination of the zinc ion is square pyramidal with two water molecules in the remaining positions (Fig 2). In contrast, in both human and yeast DPP III, zinc exhibits a tetrahedral coordination with a single water molecule that is considered to be important for the generally accepted catalytic mechanism involving water activation by Glu461 [11]. It is interesting that in the case of *Bt*DPP III, the corresponding Glu449 does not point toward either of the two zinc-coordinated water molecules. The function of this glutamic acid in the previously proposed catalytic mechanism for DPP III is to activate the water molecule that is bound to the zinc ion for the nucleophilic attack on the peptide bond of the substrate [11].

**Fig 3:**
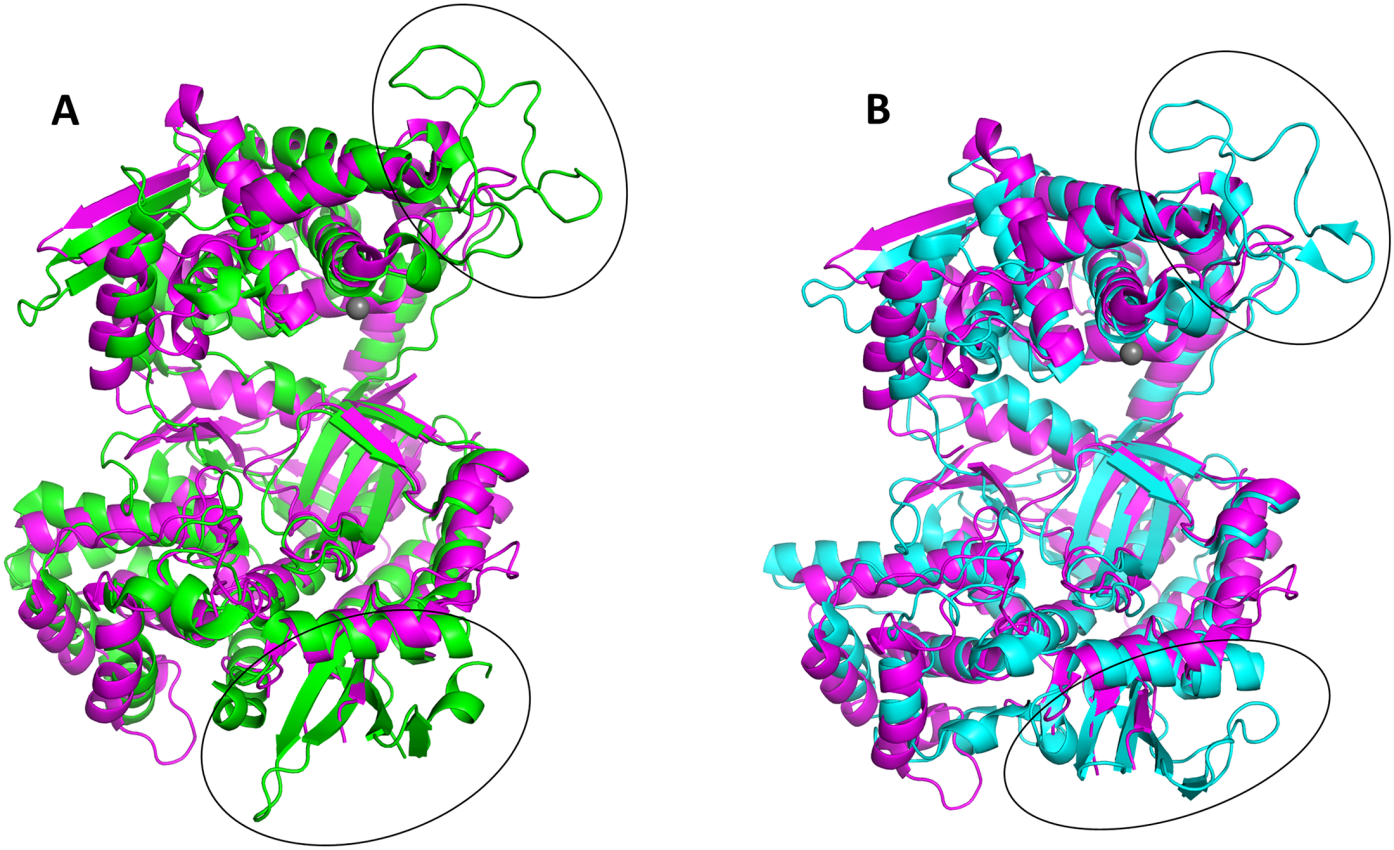
Superposition of ligand-free *Bt*DPP III on human and yeast proteins. The upper and lower domains were separately superimposed to the corresponding domains of human (A) and yeast (B) DPP III. Their structures are shown in cartoon representation: bacterial in magenta, human in green, and yeast in cyan. The black ellipses indicate the areas of the two main differences between the superimposed structures. The figure was prepared using the PyMol program (http://www.pymol.org/).

The superimposed protein backbones of ligand-free bacterial and ligand-free human DPP III structures gave rise to an RMSD value of 4.41 Å. This high RMSD value is a consequence of a difference in cleft size between human and bacterial DPP III. Namely, human DPP III is more open than the bacterial enzyme. Using the PyMol software, *Bt*DPP III was divided into an upper and a lower domain, which were subsequently treated as separate objects. Superposition of the upper domain of *Bt*DPP III (amino acids 328–364 and 404–626) with the corresponding upper domain of the human enzyme gave rise to an RMSD value of 3.0 Å. An analogous alignment of the respective lower domains of the bacterial (amino acids 24–327, 365–403 and 627–675) and human enzymes yielded an RMSD value of 2.0 Å. This method of comparison thus revealed a conservation of tertiary structures in a manner that is significantly clearer than a simple superposition of the entire proteins (Fig 3). Furthermore, a structural comparison revealed two significant differences. The loop in the upper structural domain between the two active-site motifs involved in zinc binding is 30 amino acids shorter in the bacterial protein compared to its human counterpart. In the lower domain, there is a two-stranded β-sheet (221–242, 21 residues), whereas in the human DPP III a four-stranded β-sheet and an extra α-helix (197–251, 54 residues) are found (Fig 3A). The difference in length between the *Bt*DPP III (675 amino acids) and human DPP III (737 amino acids) is 62 amino acids and corresponds to the difference in length between these two regions. Due to the high structural similarity between human and yeast enzymes (RMSD 1.36 Å), the same differences between bacterial and yeast DPP III are observed (Fig 3B).

**Fig 4:**
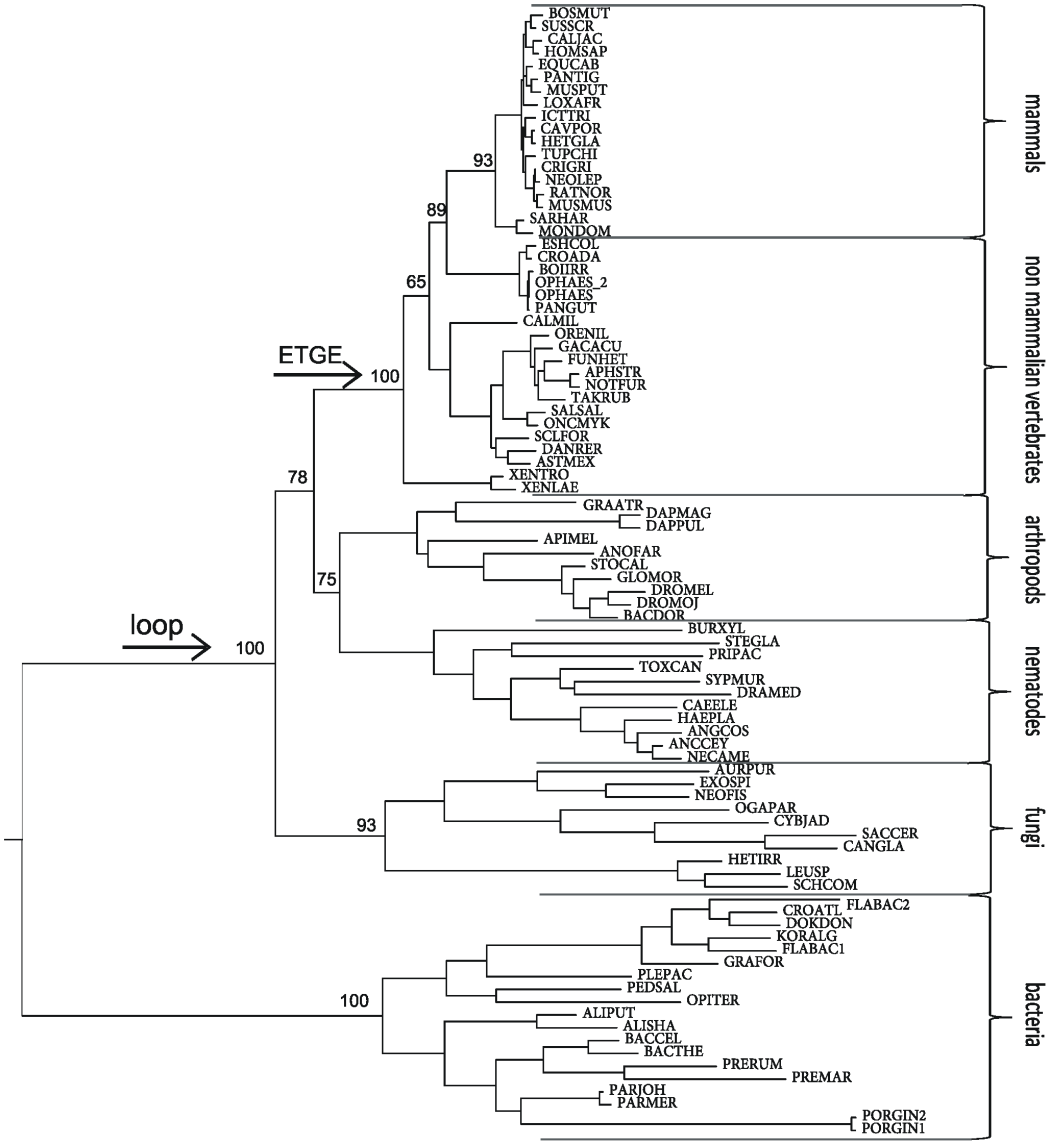
Phylogenetic tree of the M49 family. The tree is based on a multiple sequence alignment and maximum likelihood analysis of 87 peptidases of the M49 family. The branch support values are indicated at the major branch points. Species abbreviations are given in S2 Table. Arrows represent the appearance of the loop between the two conserved active-site motifs in the upper domain and the emergence of the ETGE motif.

**Fig 5:**
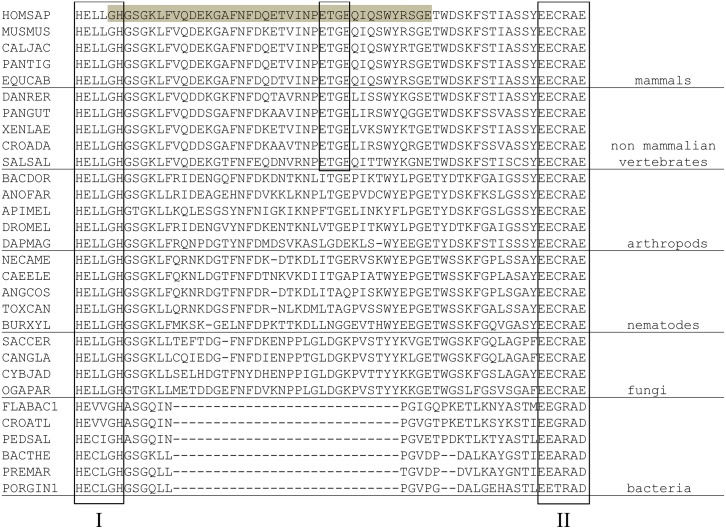
A section of a multiple sequence alignment of M49 peptidases. Thirty M49 peptidases were selected from different eukaryotic and bacterial species. The active-site motifs I and II as well as ETGE motif are framed. The loop between the two conserved active-site motifs of the human M49 peptidase is highlighted in grey. The full names of the species are given in S2 Table.

We reported earlier, based on the primary structure analysis of the M49 family members, that the length of the spacer region between the two evolutionarily conserved active-site motifs is much shorter in bacterial proteins, compared to eukaryotic DPPs III [10]. From our structural studies, it is now obvious that this region comprises an additional loop in human and yeast DPP III. In this loop, the human enzyme contains the so-called E^480^TGE motif, which is considered important for protein interaction with Keap1 and required for the activation of the Keap1-Nrf2 signalling pathway [8]. Thus, we performed a phylogenetic analysis of the M49 family of enzymes to determine the emergence of the loop between the two active-site motifs and of the ETGE motif within this loop, respectively. The obtained phylogenetic tree, constructed based on a multiple sequence alignment of 87 selected sequences, is consistent with conventional species evolution (Fig 4). As can be seen from Figs 4 and 5, the insertion between the two active-site motifs occurred before the fungi/metazoa split. However, the ETGE motif is conserved in vertebrate homologues only, and it was not found in invertebrates, fungi, or bacteria (Fig 5). Interestingly, Gacesa *et al*. reported that the Keap1-Nrf2 pathway evolved after the eukarya separated from the prokarya, but prior to the fungal-metazoan split [36]. This suggests that DPP III’s moonlighting activity, *i*.*e*. the modulatory effect on the Keap1-Nrf2 pathway, evolved much later, when the Keap1-Nrf2 pathway was already present.

It has recently been shown that yeast DPP III does not prefer the canonical synthetic substrate of mammalian DPP III, Arg-Arg-arylamide [37]. In addition, it was reported that Asp496 situated in the S2 subsite of the human enzyme is an important determinant in the selectivity of human DPP III for Arg-Arg-2-naphthylamide (Arg_2_-2NA) [38]. The superposition of the crystal structures of *Bt*DPP III and hDPP III reveals that the residue that structurally corresponds to Asp496 is Asp465 in *Bt*DPP III. Therefore, to investigate experimentally whether the bacterial enzyme shows preference for diarginyl arylamide, we determined the kinetic parameters of *Bt*DPP III for the hydrolysis of three dipeptidyl naphthylamide substrates: Arg_2_-2NA, Ala-Arg-2NA, and Phe-Arg-2NA (Table 2), which had previously been shown to be useful in discriminating between the substrate specificities of human and yeast DPP III [37]. Thus, we confirmed experimentally that *Bt*DPP III also shows a preference for Arg_2_-2NA with an 8- and 29-fold higher catalytic efficiency (*k*_cat_/*K*_m_) for this substrate than for Ala-Arg-2NA and Phe-Arg-2NA, respectively. In the yeast enzyme, Gly505 is the structural counterpart of human Asp496. In our recent study, a replacement of Gly505 with Asp yielded a protein variant of yeast DPP III which was selective for Arg_2_-2NA [37].

**Table 2:** Kinetic analysis of wild-type *Bt*DPP III^a^.

Substrate	*K*_m_ (μM)	*k*_cat_ (s^-1^)	*k*_cat_/*K*_m_ (mM^-1^·s^-1^)
Arg_2_-2NA	0.359 ± 0.030	0.384 ± 0.009	1070
Ala-Arg-2NA	3.87 ± 0.20	0.537 ± 0.008	139
Phe-Arg-2NA	4.85 ± 0.37	0.178 ± 0.004	36.8

^a^The kinetic parameters were determined from the initial reaction rates at 25°C, as described under “Materials and methods”. *K*_m_ and *k*_cat_ values are the averages of three determinations ± SD.

### X-ray structure of *Bt*DPP III in the closed conformation

For human DPP III, it was shown that a closed conformation is favoured upon ligand binding to the lower structural domain accompanied by a large structural domain movement [12, 39]. To verify if this conformational change is also characteristic for bacterial DPP III, we crystallized the Cys-null variant in the presence of the competitive inhibitor Tyr-Phe-NHOH. The solved crystal structure revealed that the protein is in the closed conformation. This dipeptidyl hydroxamic acid is a substrate analogue of DPP III and a potent inhibitor of the human and bacterial enzymes [13].

**Fig 6:**
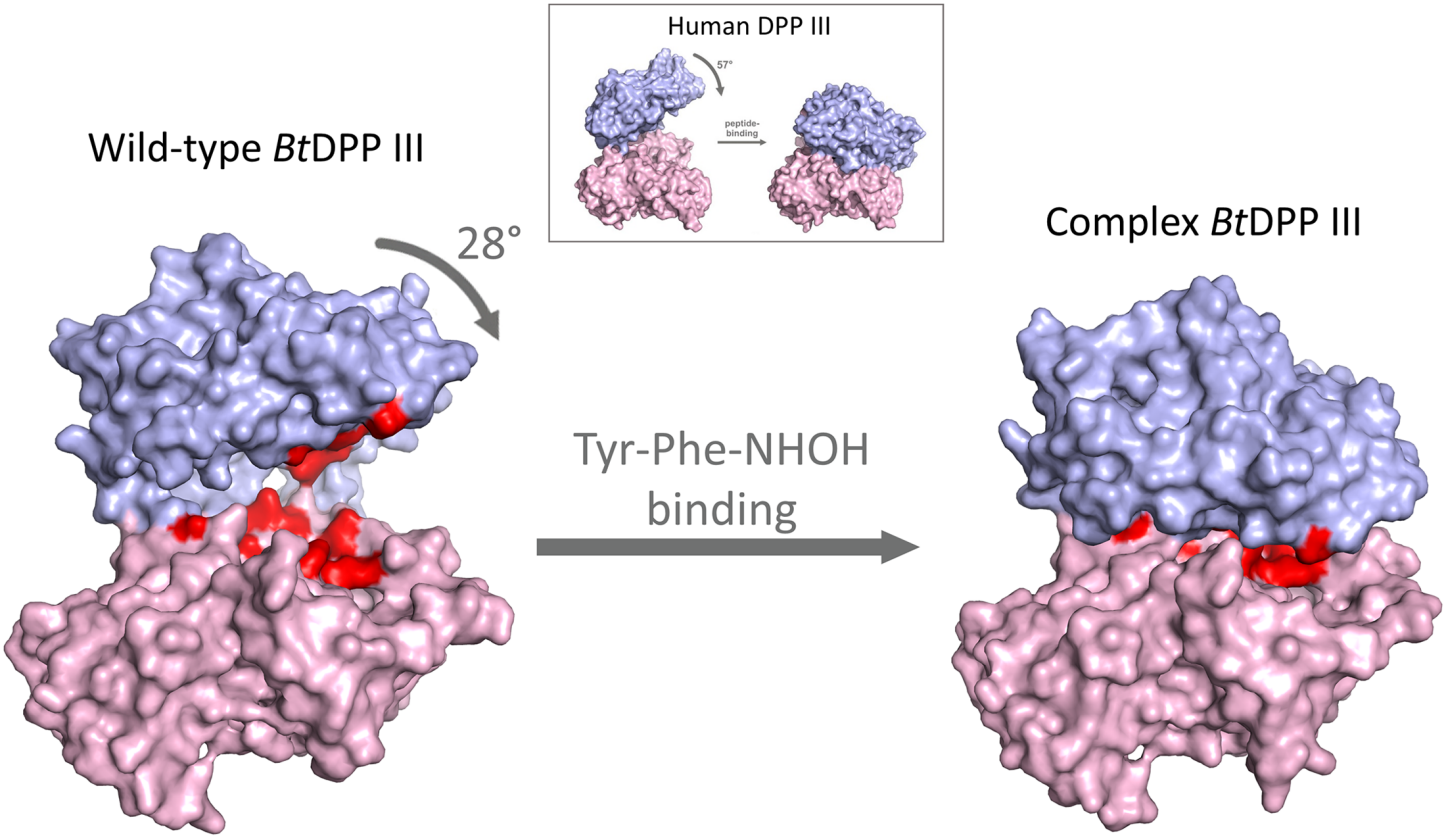
Surface representation of the ligand-free structure and the structure of the closed form of *Bt*DPP III. The upper structural domain is shown in blue and the lower one in magenta. Amino acids coloured in red are: zinc binding residues (His448, His453, and Glu476), glutamic acid essential for enzyme activity (Glu449), and structurally equivalent amino acids residues (Glu307, Tyr309, Thr380, Ile382, Gly383, Asn385, Asn388, Asp465, His533, and Tyr627) that were shown to interact with peptide substrates in human DPP III. The figure was prepared using the PyMol program (http://www.pymol.org/). The illustration showing domain movement in human DPP III, given in the square, was taken from Bezerra *et al*. [12].

In contrast to ligand-free bacterial DPP III (open form), in the presence of the inhibitor, the closed conformation was obtained, with the binding cleft closed (Fig 6). According to the DynDom program [40, 41], this conformational change can be described by a 28° rotation of one structural domain relative to the other. A superposition of the backbone atoms of the upper and lower domains of the wild-type and the structure in the closed form results in RMSD values of 0.41 and 0.26 Å, respectively. This indicates the absence of large conformational changes within the domains. According to the DynDom program, the bending regions are 330–334, 357–366, 414–418, and 618–623. Our results are in agreement with the data reported for human DPP III, where a large domain movement and formation of the closed conformation was noticed for the first time [12]. Compared to the human enzyme, a smaller rotation of domains (28° *vs*. 57°) was observed in *Bt*DPP III in the closed conformation (Fig 6).

**Fig 7:**
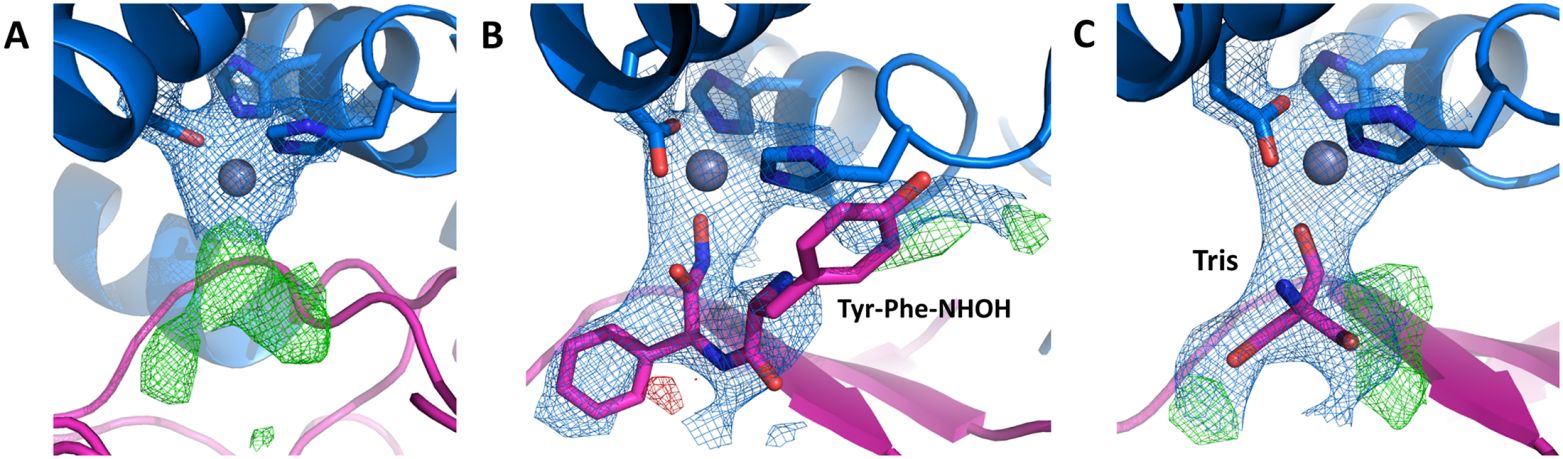
Overall cartoon representation of the active site in *Bt*DPP III structure in the closed form. 2m*F*_o_-*DF*_c_ electron density at 1 σ (blue) and *mF*_o_-*DF*_c_ electron density at 3 σ (green) correspond to the substrate-binding position. The upper structural domain is shown in blue, the lower one in magenta, and the zinc atom as a grey sphere. The amino acids binding the zinc ion, Tris, and Tyr-Phe-NHOH are shown in stick representation. (A) Electron density map in the active site; (B) electron density with Tyr-Phe-NHOH included in the refinement; (C) electron density with Tris included in the refinement. The figure was prepared using the PyMol program (http://www.pymol.org/).

The zinc ion in the active site is coordinated by His448, His453 and Glu476, as in the open form. Instead of the two water molecules coordinated to the zinc ion in the ligand-free structure, in the closed form we observed a large undefined electron density (Fig 7A). Since the protein in the closed form was obtained by cocrystallization with the inhibitor Tyr-Phe-NHOH, we tried to fit this molecule into the electron density map. However, after several cycles of refinement and employment of different orientations of the inhibitor, we realised that electron density was too small to fit the whole inhibitor molecule (Fig 7B). Even by assuming only partial occupancy of the inhibitor, we did not obtain a satisfying result. In the crystal structure of the Cys-null variant (open form), one Tris molecule, contained in the storage buffer, was bound to the zinc ion. As the same protein solution was used to obtain the structure in the closed form, we also tried to fit a Tris molecule in the undefined electron density. After several cycles of refinement, however, there was still extra electron density around the Tris molecule (Fig 7C). All other molecules present in the crystallization solution (sodium cacodylate and ammonium sulphate) were also too small to occupy the undefined electron density. As the resolution of the structure in the closed form is only 3.3 Å, and the electron density is not well-defined, we cannot determine the source of the observed electron density near the zinc ion.

Five evolutionarily conserved regions in M49 peptidases are embedded in a stretch of about 300 amino acids [10] and situated on both the lower (region 1 and region 2) and the upper protein domains (regions 3 and 4, comprising zinc-binding motifs, and region 5) [38]. S1 Fig illustrates conserved regions on the *Bt*DPP III structure. The active site of DPP III consists of the active-site zinc ion, the zinc-binding site and the substrate binding site [11, 12]. It is formed by the constituents of all five conserved regions of the M49 family, *i*.*e*. by the amino acid residues from the lower and the upper protein domains [12]. It was shown that the peptide substrate is completely buried between the two protein domains (lobes) of human DPP III [12, 39].

Even before the crystal structure of human DPP III ligand complex was resolved, our observation that Cys176, a residue from the lower domain, quite distant from the active centre (44 Å apart from the catalytic zinc ion in the crystal structure of ligand-free human DPP III), is responsible for the fast inactivation by the organomercurial compound, provided the evidence that the active site of human DPP III comprises both protein domains, which in the active form of the enzyme need to be in close contact [42].

Considering the above-mentioned evidence, it could be predicted that DPP III in the open conformation does not have catalytic activity.

Our data on the open and closed conformation of *Bt*DPP III indicate that large protein flexibility might be conserved in the M49 family. Furthermore, these results suggest that ligand binding to the prokaryotic orthologue, similarly to its human counterpart, might induce a large domain motion and the formation of a closed active site, which was previously reported to be a prerequisite for the catalytic activity of these metallopeptidases.

**Fig 8:**
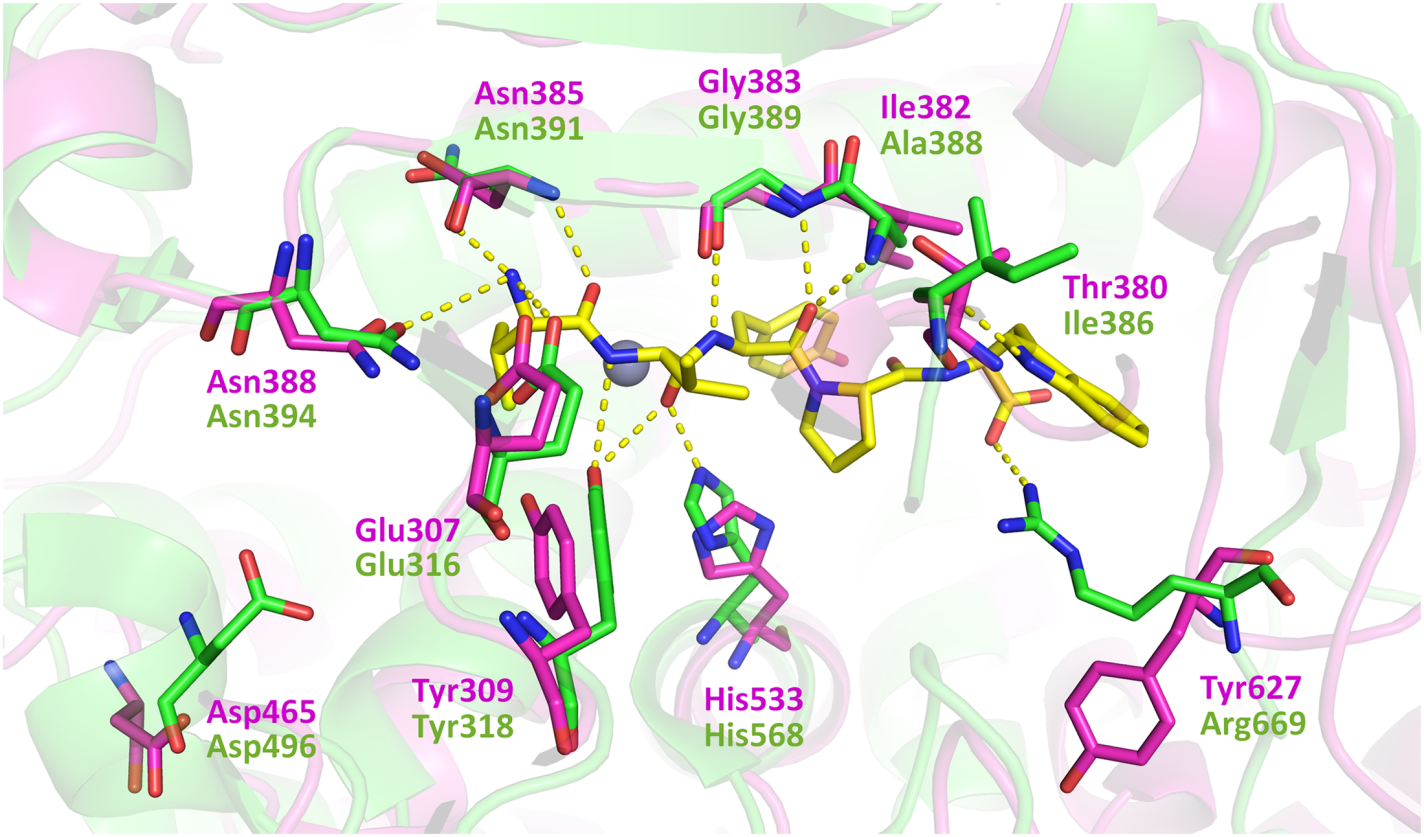
Superposition of the active sites of *Bt*DPP III and hDPP III with the bound tynorphin. The human DPP III ligand (tynorphin, VVYPW) is shown in yellow. The amino acids that make polar interactions with the peptide substrate (dashed lines) and the conserved Asp496 that is important for the substrate specificity are shown as stick models (hDPPIII and *Bt*DPPIII in green and magenta, respectively). The figure was prepared using the PyMol program (http://www.pymol.org/).

Recently, Kumar *et al*. have solved the crystal structures of the inactive E451A variant of human DPP III complexed with three opioid peptides (Met- and Leu-enkephalin, endomorphin-2), as well as with the vasoconstrictor octapeptide angiotensin II [39]. They confirmed the previously reported peptide inhibitor (tynorphin) binding mode and the large domain motion of the human enzyme upon ligand binding [12]. Based on the analysis of enzyme-substrate interactions in these structures, these authors concluded that the general peptide binding mode of human DPP III comprises extensive polar contacts of the N-terminal peptide residues and the formation of β-type interactions with the core of the enzyme [39]. We compared the crystal structures of bacterial and human DPP III and found that most amino acid residues that interact with tynorphin in the human enzyme were structurally conserved in *Bt*DPP III, suggesting that bacterial DPP III also has the potential to interact with oligopeptides. The exceptions are: Ile386, Ala388, and Arg669 that correspond to Thr380, Ile382 and Tyr627, respectively, in *Bt*DPP III (Fig 8). In yeast DPP III, as opposed to the human enzyme, only one amino acid residue from those interacting with tynorphin is not structurally conserved: Gly505 corresponding to Asp496 (not shown).

## Conclusions

We solved the crystal structures of DPP III (metallopeptidase of the M49 family) from the human gut symbiont *B*. *thetaiotaomicron*. These structures revealed a two-domain protein that exists in an open (ligand-free) and a closed conformation. The overall protein fold is, despite a low sequence similarity, very similar to that of the human and yeast orthologues. However, some significant structural differences were observed in both domains. The loop in the upper structural domain between the two active-site motifs involved in zinc binding is much (30 amino acids) shorter in the bacterial protein. By using a phylogenetic analysis, we have shown that this long insertion between the zinc binding motifs occurred before the fungal-metazoan split, and that only vertebrate homologues contain the ETGE motif, considered important for the interaction with the Keap1 protein and for the activation of the Keap1-Nrf2 signalling pathway.

The comparison of *Bt*DPP III with its human counterpart further revealed Asp465 as the residue corresponding to Asp496 of hDPP III, which is proven as the structural determinant of the human enzyme substrate selectivity for diarginyl-arylamide [38]. Our data on the open and closed conformations of *Bt*DPP III indicate that large protein flexibility might be conserved in the M49 family.

## Supporting information

S1 Table Primers used for cloning. (PDF)

S2 Table M49 family peptidases from different species used in phylogenetic analysis. (PDF)

S1 Fig Conserved regions in *Bt*DPP III. Five evolutionarly conserved regions of the M49 family are presented as red areas and correspond to: G^304^FTESYGDPLGVKASWESLV^323^ (R1), G^383^INLPNANWIRAHHGSKSVTIGNI^406^ (R2), H^448^ECLGHGSGKL^458^ (R3), E^475^EARAD^480^ (R4), and E^531^AHMRNRQLI^540^ (R5). The zinc ion is presented as a grey sphere. The figure was prepared using the PyMol program (http://www.pymol.org/).
(TIF)

S2 Fig Michaelis-Menten plots for the hydrolysis of dipeptidyl-2-naphthylamides catalysed by wild-type *Bt*DPP III. (TIF)
