# Supplementary material for: Crystal structure of dipeptidyl peptidase III from the human gut symbiont Bacteroides thetaiotaomicron
Source: PLoS One. 2017 Nov 2;12(11):e0187295. doi: 10.1371/journal.pone.0187295 (PMC5667867; doi:10.1371/journal.pone.0187295)
Supplement: S1 Table — (PDF) [file pone.0187295.s001.pdf]

Supplementary Table 1. Primers used for cloning and mutagenesis.

	Forward	Reverse
WT	5'-GCACTAGCTAGCATGGCAGTTACCGCTACGATCC-3'	5'-CCGGCTCGAGATTATTTACGGAAGGCAACGGAGAA-3'
C11S	5'-GCTACGATCCTTGCATCCTCTGGTGGGGCCAAAACAAC-3'	5'-GTTGTTTTGGCCCCACCAGAGGATGCAAGGATCGTAGC-3'
C158S	5'-CAGACTGCCGAACAACTCTCTGATGAGCTCTTCCCGG-3’	5'-CCGGGAAGAGCTCATCAGAGAGTTGTTCGGCAGTCTG-3'
C189S	5'-CTTGGTGCTGACTTCTGCCTCCAATTATTACGACGGAG-3'	5'-CTCCGTCGTAATAATTGGAGGCAGAAGTCAGCACCAAG-3'
C425S	5'-GGATTCAATGAAGAGTTTGTCTCCAATGACGAAGAGAGACAG-3'	5'-CTGTCTCTCTTCGTCATTGGAGACAAACTCTTCATTGAATCC-3'
C450S	5'-CAGACTTACATGAAAGCCTGGGACATGGTTCGG-3'	5'-CCGAACCATGTCCCAGGCTTTCATGTAAGTCTG-3'
