# Supplementary material for: Crystal structure of dipeptidyl peptidase III from the human gut symbiont Bacteroides thetaiotaomicron
Source: PLoS One. 2017 Nov 2;12(11):e0187295. doi: 10.1371/journal.pone.0187295 (PMC5667867; doi:10.1371/journal.pone.0187295)
Supplement: S2 Table — (PDF) [file pone.0187295.s002.pdf]

Supplementary Table 2. M49 family peptidases from different species used in phylogenetic analysis.

**Abbreviation**	**Species**	**Accession number**	**Species group**
MONDOM	*Monodelphis domestica*	F6ZXH8	mammals
SARHAR	*Sarcophilus harrisii*	G3W555	
MUSMUS	*Mus musculus*	Q99KK7	
RATNOR	*Rattus norvegicus*	O55096	
NEOLEP	*Neotoma lepida*	A0A1A6HW94	
CRIGRI	*Cricetulus griseus*	G3I2H0	
TUPCHI	*Tupaia chinensis*	L8Y5A0	
HETGLA	*Heterocephalus glaber*	A0A0P6JC08	
CAVPOR	*Cavia porcellus*	H0UUH1	
ICTTRI	*Ictidomys tridecemlineatus*	I3ME44	
LOXAFR	*Loxodonta africana*	G3T8L7	
MUSPUT	*Mustela putorius*	M3XVE8	
PANTIG	*Panthera tigris*	A0A060KZU3	
EQUCAB	*Equus caballus*	F6TNY4	
HOMSAP	*Homo sapiens*	Q9NY33	
CALJAC	*Callithrix jacchus*	U3FHY1	
SUSSCR	*Sus scrofa*	L8I2E6	
BOSMUT	*Bos mutus*	F1RU52	
PANGUT	*Pantherophis guttatus*	A0A098LYJ3	non mammalian vertebrates
OPHAES	*Opheodrys aestivus*	A0A098LWX2	
BOIIRR	*Boiga irregularis*	A0A0B8RRY3	
CROADA	*Crotalus adamanteus*	J3RZ30	
ESHCOL	*Echis coloratus*	A0A0A1WDL0	
XENLAE	*Xenopus laevis*	Q6DE90	
XENTRO	*Xenopus tropicalis*	F7DZ42	
ASTMEX	*Astyanax mexicanus*	W5KIP6	
DANRER	*Danio rerio*	Q6DI20	
SCLFOR	*Scleropages formosus*	A0A0N8JZV5	
ONCMYK	*Oncorhynchus mykiss*	A0A060X8W4	
SALSAL	*Salmo salar*	B5X435	
TAKRUB	*Takifugu rubripes*	H2TEX3	
NOTFUR	*Nothobranchius furzeri*	A0A1A8ABL4	
APHSTR	*Aphyosemion striatum*	A0A1A7YL47	
FUNHET	*Fundulus heteroclitus*	A0A146P4W4	
GASACU	*Gasterosteus aculeatus*	G3QAW5	
ORENIL	*Oreochromis niloticus*	I3JPG4	
CALMIL	*Callorhinchus milii*	V9KGU9	
BACDOR	*Bactrocera dorsalis*	A0A034VA64	arthropods
DROMOJ	*Drosophila mojavensis*	B4K999	
DROMEL	*Drosophila melanogaster*	Q9VHR8-2	
GLOMOR	*Glossina morsitans*	D3TMQ7	
STOCAL	*Stomoxys calcitrans*	A0A1I8NZT2	
ANOFAR	*Anopheles farauti*	A0A182QC05	
APIMEL	*Apis mellifera*	A0A088A023	

DAPPUL	*Daphnia pulex*	E9GTX0	
DAPMAG	*Daphnia magna*	A0A162P8G7	
GRAATR	*Graphocephala atropunctata*	A0A1B6KD94	
NECAME	*Necator americanus*	W2SLT7	nematodes
ANCCEY	*Ancylostoma ceylanicum*	A0A0D6M7T1	
ANGCOS	*Angiostrongylus costaricensis*	A0A0R3PGV3	
HAEPLA	*Haemonchus placei*	A0A0N4X8W6	
CAEELE	*Caenorhabditis elegans*	G5ECW7	
DRAMED	*Dracunculus medinensis*	A0A0N4U2V0	
SYPMUR	*Syphacia muris*	A0A0N5AXJ5	
TOXCAN	*Toxocara canis*	A0A0B2VIP3	
PRIPAC	*Pristionchus pacificus*	A0A0F5CUX7	
STEGLA	*Steinernema glaseri*	A0A1I7YLE5	
BURXYL	*Bursaphelenchus xylophilus*	A0A1I7SCL0	
SCHCOM	*Schizophyllum commune*	D8PVF6	fungi
LEUSP	*Leucoagaricus sp*	A0A137QPX9	
HETIRR	*Heterobasidion irregulare*	W4KKQ6	
CANGLA	*Candida glabrata*	A0A0W0CQ92	
SACCER	*Saccharomyces cerevisiae*	Q08225	
CYBJAD	*Cyberlindnera jadinii*	A0A0H5CH85	
OGAPAR	*Ogataea parapolymorpha*	W1QG85	
NEOFIS	*Neosartorya fischeri*	A1CY04	
EXOSPI	*Exophiala spinifera*	A0A0D1Y698	
AURPUR	*Aureobasidium pullulans*	A0A074XX23	
FLABAC2<br>KORALG<br>DOKDON<br>CROATL<br>GRAFOR<br>FLABAC1<br><br>OPITER<br><br>PEDSAL<br>PLEPAC<br>BACCEL<br><br>BACTHE<br>PARMER<br>PARJOH<br><br>PREMAR<br>PRERUM<br><br>ALISHA<br>ALIPUT<br>PORGIN1<br>PORGIN2	*Flavobacteriales bacterium*<br>*Kordia algicida*<br>*Dokdonia donghaensis*<br>*Croceibacter atlanticus*<br>*Gramella forsetii*<br>*Flavobacteria bacterium* (strain BBFL7)<br>*Opitutus terrae* (strain DSM 11246/PB90-1)<br>*Pedobacter saltans* DSM 12145<br>*Plesiocystis pacifica* SIR-1<br>*Bacteroides cellulosilyticus* DSM 14838<br>*Bacteroides thetaiotaomicron*<br>*Parabacteroides merdae* ATCC 43184<br>*Parabacteroides johnsonii* DSM 18315<br>*Prevotella marshii* DSM 16973<br>*Prevotella ruminicola* (strain ATCC 19189/JCM 8958/23)<br>*Alistipes shahii* WAL 8301<br>*Alistipes putredinis* DSM 17216<br>*Porphyromonas gingivalis*<br>*Porphyromonas gingivalis* (strain ATCC 33277/ DSM 20709/JCM 12257)	A4AQA4<br>A9ECI1<br>A2TUF5<br>A3U8C5<br>A0LXF2<br>Q26C69<br><br>B1ZZ02<br><br>E4KIB0<br>A6GID1<br>E2NMV7<br><br>Q8A6N1<br>A7ACL0<br>B7BE50<br><br>E0NQE0<br>D5ERS0<br><br>D4IQ41<br>B0MWP5<br>Q7MX92<br>B2RLB9	bacteria
