# Supplementary figures and images for: Crystal structure of dipeptidyl peptidase III from the human gut symbiont Bacteroides thetaiotaomicron
Source: PLoS One. 2017 Nov 2;12(11):e0187295. doi: 10.1371/journal.pone.0187295 (PMC5667867; doi:10.1371/journal.pone.0187295)

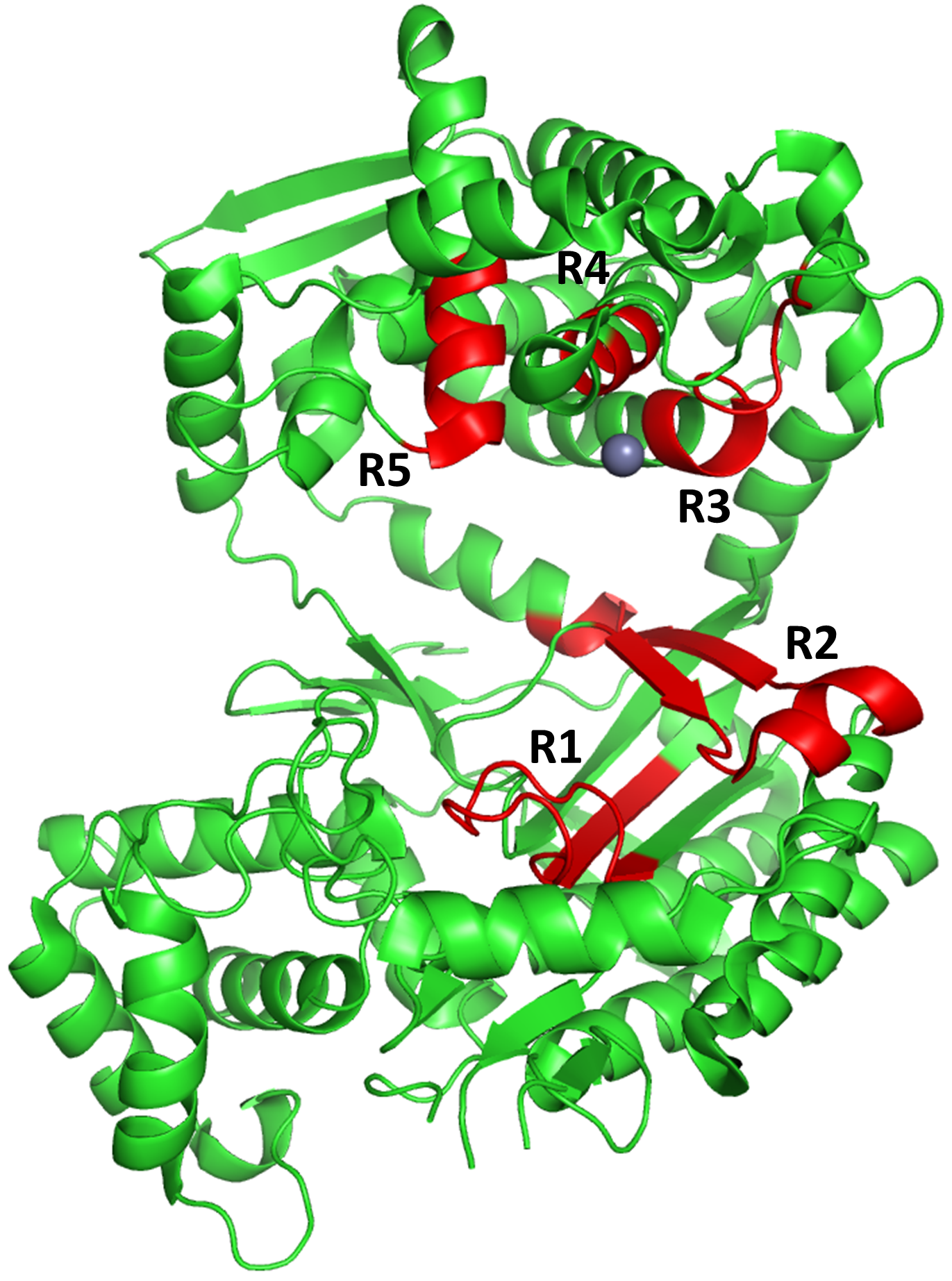

Supplement: S1 Fig — Five evolutionarly conserved regions of the M49 family are presented as red areas and correspond to: G304FTESYGDPLGVKASWESLV323 (R1), G383INLPNANWIRAHHGSKSVTIGNI406 (R2), H448ECLGHGSGKL458 (R3), E475EARAD480 (R4), and E531AHMRNRQLI540 (R5). The zinc ion is presented as a grey sphere. The figure was prepared using the PyMol program (http://www.pymol.org/). (TIF) [file pone.0187295.s003.tif]

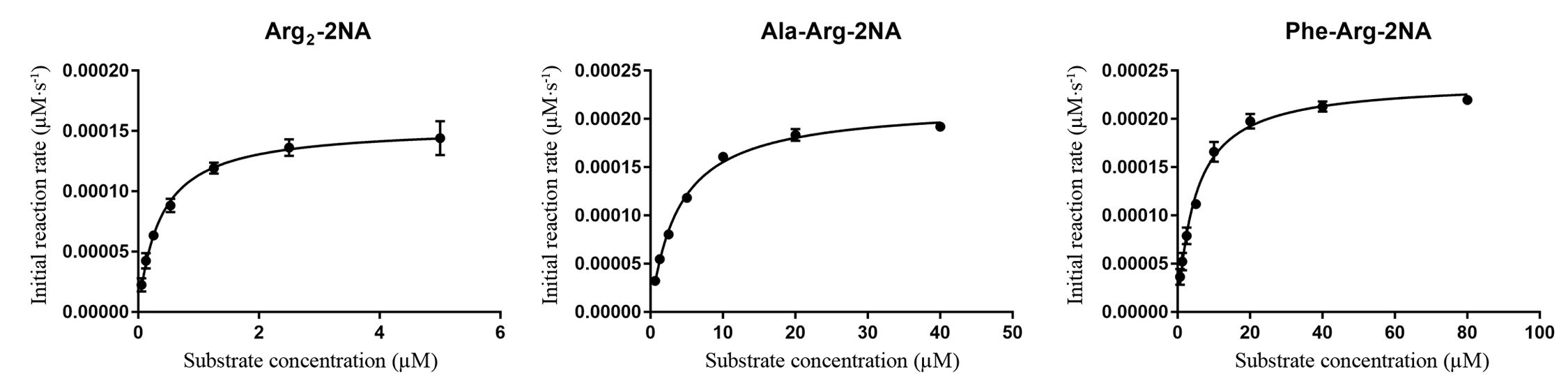

Supplement: S2 Fig — (TIF) [file pone.0187295.s004.tif]
